# Does Temperament Constitute a Risk Factor for Adult Attention Deficit Hyperactivity Disorder?

**DOI:** 10.7759/cureus.70915

**Published:** 2024-10-06

**Authors:** Gonca Ayse Unal

**Affiliations:** 1 Psychiatry, Mersin Sehir Egitim ve Arastirma Hastanesi, Mersin, TUR

**Keywords:** anxiety, attention deficit hyperactivity disorder, cyclothymic disorder, irritable mood, temperament

## Abstract

Objective: The aim of this study was to examine the temperament characteristics of adults with attention deficit hyperactivity disorder (ADHD) and the relationship between ADHD subtypes and temperament. Additionally, the study aimed to investigate the relationship between childhood ADHD symptoms and temperament.

Methods: The study included 59 ADHD patients aged between 18 and 60 years and 44 healthy controls. All participants completed the Wender-Utah Rating Scale (WURS) and the Adult Attention Deficit Hyperactivity Disorder Diagnosis and Rating Scale. Temperament characteristics were assessed using the Temperament Evaluation of Memphis, Pisa, Paris, and San Diego-Autoquestionnaire scale (TEMPS-A).

Results: The ADHD group had significantly higher scores for cyclothymic, irritable, and anxious temperament compared to the control group (p<0.001). The number of individuals with cyclothymic, irritable, and anxious temperament was also significantly higher in the ADHD group (p=0.007, p=0.018, p=0.029, respectively). Positive correlations were found between cyclothymic and depressive temperament scores and WURS scores (r=0.278, p=0.033; r=0.326, p=0.012, respectively), between hyperthymic temperament scores and hyperactivity scores (r=0.399, p=0.002), and between depressive temperament scores and attention deficit scores (r=0.303, p=0.020). There was no relationship between ADHD subtypes and dominant temperament (p>0.05).

Conclusion: The most common dominant temperament in the ADHD group was cyclothymic, irritable, and anxious. The positive correlation between WURS scores and cyclothymic temperament suggests that cyclothymic temperament may be a risk factor for adult ADHD.

## Introduction

In the realm of psychosocial research, temperament is thought to be an inherited and developmentally stable personality component [1]. Temperament is a set of relatively stable, mainly biologically based traits that influence how a person reacts to their environment. Temperamental traits are stable from childhood to adulthood and are structurally consistent across different cultures and ethnicities [2]. The temperament type is determined by genetic factors, and temperament traits are estimated to be nearly 50% heritable [3]. Affective temperaments that are defined by Akiskal et al. [3] are considered structures determined jointly by heredity, developmental factors, and facilitating factors. Kraepelin indicated that affective temperaments can remain as character traits without turning into an affective illness throughout the life of the individual or can be the beginning of an episodic illness [4]. Akiskal et al. [5] created original criteria for five affective temperaments, including cyclothymic, dysthymic, hyperthymic, irritable, and anxious, and developed the self-report scale Automatic Questionnaire Version for Temperament Assessment of Memphis, Pisa, Paris, and San Diego (TEMPS-A) to evaluate these temperaments. Affective temperament screening plays a crucial role in the diagnosis of psychiatric disorders, as temperaments can be determinants of health and functional impairment, in addition to comorbidities and the severity of the disorder. Owing to the high comorbidity rates of ADHD with bipolar disorder, the researchers searched clinical and biological overlaps between the disorders and put forward that the determination of temperament in ADHD may result in better treatment outcomes. Because of these reasons, the determination of affective temperament is important for ADHD as well [6].

Attention deficit hyperactivity disorder (ADHD) is defined as a chronic, multifactorial, and heterogeneous disorder, which is qualified by symptoms of inattention, hyperactivity, and impulsivity [7]. ADHD is the most widespread neurodevelopmental disorder in childhood and affects approximately 5-8% of kids [8]. Two-thirds of individuals diagnosed with ADHD in childhood have continuity of symptoms into adulthood, with an estimated prevalence of 3-5% worldwide for affected adults [9]. Furthermore, individuals with ADHD often have emotional dysregulation such as irritability, anger, frequent mood swings, and difficulty regulating behavior in response to emotional activation [10].

According to Cloninger's model, character maturation is severely impaired in childhood-onset neuropsychiatric disorders, and personality disorders may occur as a result. Neurocognitive skills such as attention, impulse control, empathy, and communication are crucial for personality development [11]. Attention problems can lead to difficulties in important neurocognitive functions, such as perceiving the environment, understanding social cues, and empathizing with others. Therefore, ADHD can be a significant barrier to personality development [11].

Individuals with ADHD often experience mood changes that last from hours to several days, similar to individuals with cyclothymic temperament. These individuals may experience internal mood changes that result in brief sub-syndrome cycles of depression or hypomania [12]. Despite this, to our knowledge, it is hard to find a lot of studies based on the emotional temperament characteristics of adults diagnosed with ADHD. Using the TEMPS-A, a review identified six studies, with three additional studies published afterward [6,13-15]. In this review, the results were coherent with significant similarity between ADHD and depressive, cyclothymic, anxious, and irritable temperaments [6].

In one of three studies, it has been reported that individuals with ADHD have higher scores than those of controls in all scales of TEMPS-A (except for the hyperthymia scale) [13]. In the study, temperament was appraised via scale scores, and dominant temperament was not taken into consideration [13]. In the other two studies, only the cyclothymic scale of the TEMPS-A was used, and other temperaments were not explored [14,15]. Only one study in the literature has examined the relationship between adult ADHD subtypes and temperament [14]. However, this study only looked at the connection with cyclothymic temperament and did not consider other temperaments.

In this study, the purpose was to examine the relationship between adult ADHD and its subtypes and temperament. It is believed that temperament characteristics play a role in the progression of childhood ADHD symptoms into adulthood. As a result, it was also aimed to examine the relationship between childhood ADHD symptoms and temperament. The hypothesis of this study is that childhood ADHD symptoms may be associated with depressive, cyclothymic, irritable, and anxious temperaments, which are more common in adults with ADHD, and that these temperament characteristics may be a risk factor for the continuation of ADHD in adulthood.

## Materials and methods

Study design

Before conducting the research, ethical approval was obtained from the Toros University Scientific Research and Publication Ethics Committee on January 26, 2024, and decision number 2. This study was conducted at a training and research hospital between January and May 2024. The study was conducted in accordance with the Declaration of Helsinki. As a result of the power analysis, it was calculated that 90% power would be obtained with 95% confidence when there were at least 21 patients. The patient group consisted of 59 consecutive patients diagnosed with ADHD who applied to the psychiatry outpatient clinic. The control group consisted of 44 healthy individuals, carefully selected from the hospital staff who matched in age with the patient group. The study's author, a psychiatrist, did an in-depth clinical interview with the volunteers included in the control group face-to-face. Those without mental disorders were included in the control group.

The co-occurrence of ADHD and another psychiatric diagnosis may make the diagnosis of ADHD more difficult. Therefore, ADHD patients with another psychiatric diagnosis according to DSM-5 criteria during the clinical interviews were not included in the study. Exclusion criteria for the study were having a neurological or chronic disease, mental retardation, or a psychiatric disorder due to a medical condition.

The study included patients diagnosed with adult ADHD according to the Diagnostic and Statistical Manual of Mental Disorders-V (DSM-5) criteria and healthy controls aged 18-60. Participants voluntarily took part in the study after being informed, and written informed consent was obtained from all subjects involved in the study. In the consent form, participants were informed that the study would publish articles based on information collected, but with no identifiable information. Data collection took an average of 20 minutes.

For the correct identification and diagnosis of adult ADHD, combined with DSM-5 screening criteria and the use of recommended questionnaires are recommended as best practice. In this study, the Wender Utah Rating Scale and Adult Attention Deficit Hyperactivity Disorder Diagnostic and Evaluation Scale were administered to the participants by the author of the study, who is a psychiatrist, along with clinical interviews according to DSM-5 criteria. These scales are clinical scales whose validity and reliability have been studied in Turkish and whose diagnostic sensitivity, validity, reliability, and test-retest consistency have been found to be high and are used in clinical practice. Patients who scored 36 points or more on the “Wender Utah Rating Scale” and replied with a minimum of 6 out of 9 questions as 2 or 3 points in the first and/or second parts of the “Adult Attention Deficit Hyperactivity Disorder Diagnosis and Evaluation Scale” were diagnosed with ADHD.

Temperament characteristics were evaluated using the Temperament Evaluation of Memphis, Pisa, Paris, and San Diego-Autoquestionnaire (TEMPS-A) scale. Since the person's temperament will not change throughout life, a cross-sectional study was planned and the scale was applied to both groups.

Instruments

The patients included in the study were evaluated using the Wender-Utah Rating Scale, Adult ADD/ADHD DSM IV-Based Diagnostic Screening and Rating Scale, TEMPS-A, and a social demographic data form. The social demographic data form was a data sheet developed by the researchers to study the socio-demographic features of the study groups.

The WURS is a self-report scale developed by the Utah group to assess the childhood symptoms of adults associated with ADHD [16]. Oncü et al. [17] established the Turkish validity and reliability of WURS, with a cut-off score of 36. Each item can be rated from 0-4 (0-not at all/very slightly; 1-mildly; 2-moderate; 3-quite a bit; 4-very much). Sample items include “As a child, I experienced inattentive daydreaming” and “As a child in school I did not achieve my potential”. The score is calculated as the sum of the item answers [13].

Our team chose to use the Adult ADD/ADHD DSM IV-Based Diagnostic Screening and Rating Scale (Adult Attention Deficit Hyperactivity Disorder Diagnosis and Evaluation Scale developed by Turgay in 1995. This tool is a self-screening assessment [18]. When developing the adult ADD/ADHD Scale, 18 symptoms of the diagnostic criteria in DSM-IV were reframed to make them easier for patients to understand. The first part of this scale had 9 inattention questions, and the second part had 9 hyperactivity/impulsivity questions. The most frequently associated symptoms in ADHD that were not included in the DSM-IV ADHD diagnostic criteria were included in the third part of the scale. Gunay et al. [19] reported that the Turkish form of the scale is valid and reliable. The severity and frequency of the symptoms are placed on a Likert scale with 0, 1, 2, and 3 describing “not at all,” “just a little,” “pretty much,” and “very much,” respectively. “Pretty much” and “very much” ratings are considered clinically significant. Participants who gave an answer of 2 or 3 points to at least six of the nine questions in the first and/or second parts of the Adult ADHD Diagnosis and Evaluation Scale were diagnosed with ADHD.

The TEMPS-A was developed by Akiskal et al. [5]. It is a true/false self-report scale designed to evaluate emotional temperament traits present throughout one's lifetime. The Turkish version includes 99 items to describe five temperament subtypes. An individual may have one or more dominant temperaments, or may not have any dominant temperament. Items 1-18 define depressive temperament, items 19-37 define cyclothymic temperament, items 38-57 define hyperthymic temperament, items 58-75 define irritable temperament, and items 76-99 define anxious temperament. The dominant affective temperament was determined according to the cut-off scores for each affective temperament subtype, which are 13 for depressive, 18 for cyclothymic, 20 for hyperthymic, 13 for irritable, and 18 for anxious [20].

Statistical analysis

Statistical analysis of the study data was analyzed with the Statistical Product and Service Solutions (SPSS, version 24; IBM SPSS Statistics for Windows, Armonk, NY). The normal distribution of continuous variables was assessed by the Shapiro-Wilk test. Categorical variables were presented by numbers and percentages. Continuous variables that followed a normal distribution were reported as mean and standard deviation (SD). Student's T-test was used to make a comparison between the averages of two independent groups for variables conforming to normal distribution. The relationships between categorical variables were examined using chi-square analysis. Pearson correlation analysis was used to assess the correlation between the data. The statistical significance level was considered as p <0.05 for all comparisons.

## Results

The mean age of the patients with ADHD was 28.93±8.1 years, while the mean age of the control group was 32.3±8.2 years. The ADHD group consisted of 35 females and 24 males, while the control group had 29 females and 15 males. The distribution of ADHD subtypes was as follows: attention deficit subtype (28.8%), hyperactivity subtype (27.1%), and combined subtype (44.1%).

Cyclothymic, irritable, and anxious temperament scores were significantly higher in the ADHD group than in the control group (all p<0.001) (Table 1).

**Table 1:** The comparison between terms of affective temperament scores of ADHD and control groups *Student’s t-test, p < 0.005 ADHD=Attention Deficit Hyperactivity Disorder; SD=Standard Deviation

	ADHD Mean±SD	Control Mean±SD	P
Depressive Temperament	8.23±3.41	7.08±5.64	0.244
Cyclothymic Temperament	11.67±4.76	4.61±4.07	<0.001*
Hyperthymic Temperament	9.33±4.87	8.76±5.09	0.601
Irritable Temperament	6.67±4.38	2.74±3.11	<0.001*
Anxious Temperament	10.39±10.18	2.76±2.55	<0.001*

According to the dominant temperament, the number of people with cyclothymic, irritable, and anxious temperaments was significantly higher in the ADHD group than in the control group (p=0.007, p=0.018, p=0.029, respectively). Dominant depressive temperament subgroups were found with similar frequencies between groups (p>0.05, Table 2).

**Table 2:** Comparison of dominant temperament between ADHD and control groups *Chi-square analysis, p<0.005; ADHD=Attention Deficit Hyperactivity Disorder

	ADHD n (%)	Control n (%)	p
Dominant Depressive	8 (13.6)	7 (15.9)	0.738
Dominant Cyclothymic	9 (15.3)	0 (0)	0.007
Dominant Hyperthymic	0 (0)	0 (0)	
Dominant Irritable	7 (11.9)	0 (0)	0.018*
Dominant Anxious	6 (10.2)	0 (0)	0.029

In the correlation analysis between the WURS and Adult ADD/ADHD DSM-IV Diagnostic Screening and Rating Scale and temperament scores, statistically significant correlations were found between cyclothymic and depressive temperament scores. WURS scores at a weakly positive (r=0.278, p=0.033, r=0.326, p=0.012), between depressive temperament scores and attention deficit scores at a weakly positive (r=0.303, p=0.020), and between hyperthymic temperament scores, and hyperactivity scores at a weakly positive (r=0.399, p=0.002) (Table 3).

**Table 3:** Correlation of Wender-Utah, Attention Deficit and Hyperactivity Scores and Temperament scores *Pearson correlation analysis, p<0.005 There was no relationship between ADHD subtypes and dominant temperament (p>0.05). ADHD=Attention Deficit Hyperactivity Disorder

	Wender-Utah		Attention deficit		Hyperactivity	
	R	p	r	p	r	p
Depressive	0.326	0.012	0.303	0.020*	-0.256	0.076
Cyclothymic	0.278	0.033*	0.180	0.217	-0.066	0.652
Hyperthymic	-0.057	0.695	-0.157	0.280	0.399	0.002*
Irritable	0.182	0.211	-0.013	0.929	0.111	0.450
Anxious	0.249	0.084	-0.053	0.715	-0.229	0.113

## Discussion

The main finding of the present study was that cyclothymic, irritable, and anxious temperament scores were higher in the adult ADHD group. According to the dominant temperament, there were also more people with irritable, cyclothymic, and anxious dominant temperaments than in the control group. There was a positive relationship between the WURS score and cyclothymic and depressive temperament scores, between hyperactivity and hyperthymic temperament scores, and between attention deficit and depressive temperament scores.

In a review that included six studies using TEMPS-A in adult ADHD, the results were consistent with significant relationships between adult ADHD and depressive, cyclothymic, anxious, and irritable temperaments [6]. These studies are summarized in (Table 4) [6,12,21-25].

**Table 4:** Summary of six studies using TEMPS in adult ADHD in the review Table 4 adapted from [6]; ↑ indicates higher TEMPS-A scores in people with ADHD symptoms or syndromes compared with standard cut-offs; ADHD=Attention Deficit and Hyperactivity Disorder; BD=Bipolar Disorder; HC=Healthy Controls; MDD=Major Depressive Disorder

Authors/Year	Participants	TEMPS Results in ADHD
[21]	• 81 ADHD	• ↑ cyclothymic, irritable and anxious in adult ADHD than childhood ADHD
		• Scores of cyclothymic and irritable temperaments significantly associated with symptoms’ severity in adult ADHD
[22]	• 60 ADHD • 50 BD • 30 HC	• ↑ depressive, cyclothymic, irritable and anxious in ADHD patients and BD patients compared with controls
[23]	300 subjects • 100 BD •100 MDD • 100 HC Tested for ADHD symptoms	• ↑ depressive, cyclothymic, irritable, and anxious all the three groups positive for ADHD symptoms. • No significant association with hyperthymic temperament.
[24]	3280 men	Cyclothymic, Irritable and Anxious temperaments are related with occurrence and severity of ADHD Symptoms.
[25]	120 subjects • 40 ADHD • 40 BD • 40 HC	• No dominant temperament in HC • ADHD: ↑ scores on all domains (depressive, cyclothymic, irritable and anxious) except hyperthymic.
		• ↑ Irritable temperament in ADHD than BD
		• Similar results for ADHD and BD on all domains (except Irritable)
[12]	• 586 ADHD • 721 HC	TEMPS-A Scores: • 71% of ADHD patients have cyclothymic temperament, 13% of controls have cyclothymic temperament
		ADHD Patients + Cyclothymic Temperament Associations:
		• ↑ childhood and adult ADHD symptoms
		• ↑ psychiatric comorbidity (Bipolar Disorder 10%)

The most comprehensive study investigating the relationship between adult ADHD and temperament using TEMPS-A was conducted by Skala et al. [24], in which 3,280 people were evaluated. They stated that cyclothymic, irritable, and anxious temperaments are related to the occurrence and severity of ADHD symptoms [24].

Landaas et al. [12] studied 586 adults with ADHD and 721 healthy controls. Approximately 71% of ADHD patients had cyclothymic temperament, while this rate was 13% in healthy controls. In general, patients with both ADHD and cyclothymic temperament had higher childhood and adult ADHD symptoms and more frequent psychiatric comorbidities, as well as lower educational and occupational success compared to healthy controls [12]. In a study investigating the morbidity and occupational functioning of people with cyclothymic temperament in the general population, it was found that the frequency of ADHD in cyclothymics was 9.7%, while it was 3.6% in non-cyclothymics. Participants with cyclothymic temperament were also reported to have more severe childhood and current ADHD symptoms than those without [14]. Ozdemiroglu et al. [21] studied a sample of 81 participants (48 women and 33 men) diagnosed with ADHD. The participants were divided into two groups: those with childhood ADHD (n=46) and those with adult ADHD (n=35). They found that the adult ADHD group had significantly higher scores on the cyclothymic, irritable, and anxious subscales of the TEMPS-A. Cyclothymic and irritable temperament scores are significantly associated with the severity of ADHD symptoms in adulthood [21].

In the present study, consistent with the literature, cyclothymic, irritable, and anxious temperaments were found to be higher in individuals with adult ADHD. In the literature, cyclothymic temperament has been associated with childhood ADHD and the severity of symptoms. When it is considered that WURS is evaluating childhood ADHD, WURS scores were positively related to cyclothymic temperament scores in this study, suggesting that cyclothymic temperament can be a risk factor for adult ADHD. In the present study, when temperament characteristics of ADHD subtypes were examined, the relationship between attention deficit scores and depressed mood scores, and between hyperactivity scores and hyperthymic temperament scores was detected. Distractibility and difficulty maintaining attention are the most substantial symptoms of adult ADHD. These people were mostly ineffective in their lives, as it is often difficult for them to memorize schedules, manage time, make plans, finish the existing tasks, and coordinate their tasks [26]. Therefore, the attention deficit subtype is thought to have depressive temperament characteristics. Hyperthymic temperament, especially characteristics of being happy, social, self-confident, lower sleep requirement, energetic and euphoric, ready to act and being interventional, etc., is also consistent with hyperactivity [27]. Therefore, it is suggested that the hyperactivity subtype is associated with hyperthymic temperament.

Our current findings reveal that temperament traits have the possibility to take a role in making decisions that may develop a diagnosis, differential diagnosis, or treatment efficacy. In fact, studies that aim to evaluate temperament from a diagnostic, prognostic, and therapeutic perspective may be guided by this case.

Limitations

The limitations of the study were the small number of participants and the evaluation of the temperament characteristics of the patients with only one scale. There are actually different temperament assessment methods, there is not any direct comparison between these methods, and this may decrease the reliability of the results. Although the study excludes individuals with neurological or chronic diseases, it does not provide sufficient details on how potential psychiatric comorbidities such as mood disorders were screened out or managed. Comorbid conditions could significantly influence both ADHD symptoms and temperament traits, making it difficult to isolate ADHD-specific temperament patterns. Although the study group is relatively homogeneous, longitudinal and experimental studies are needed. Conducting systematic studies with larger samples in this field would be beneficial in obtaining clearer results on ADHD-temperament relationships.

Future research is necessary to investigate how temperament affects ADHD patients' pharmacological treatment response and the course of their illness and if temperament scores affect long-term therapeutic outcomes.

## Conclusions

In conclusion, in recent years, the number of studies on the relationship between ADHD and temperament traits has increased. Given the current study design, there appears to be an association between temperamental traits and ADHD. Cyclothymic, irritable, and anxious temperament was the most common dominant temperament for the ADHD group. Additionally, WURS scores were positively correlated with cyclothymic temperament scores, suggesting that cyclothymic temperament may be a risk factor for adult ADHD.
